# Supplementary material for: Plasma Lipid Profiling Contributes to Untangle the Complexity of Moyamoya Arteriopathy
Source: Int J Mol Sci. 2021 Dec 14;22(24):13410. doi: 10.3390/ijms222413410 (PMC8708587; doi:10.3390/ijms222413410)
Supplement: Supplementary file 1 [file ijms-22-13410-s001.zip › Supplementary Materials_ijms-1491031.pdf]

ID		Age	Gender	CVD type	NIHSS	U-B	Suzuki grading	Vascular risk factors	Pharmacological therapy
**MA**	**3**	43	F	IS	4	U	I	-	AG, ASA,ST, AE
**MA**	**5**	44	F	HS	5	B	IV	H	AE, AD
**MA**	**9**	54	M	TIA	0	B	IV	DL	AG, ASA,AD
**MA**	**10**	59	M	IS	4	B	IV	HoS	AG, ASA
**MA**	**11**	37	F	HS	0	U	IV	HT	AG, ASA
**MA**	**14**	15	F	TIA	0	U	IV	-	AG, ASA
**MA**	**15**	47	F	IS	8	B	IV	H, PI,	AG, ASA, ST, AE
**MA**	**19**	42	F	IS	2	B	IV	HT	AG, ASA, ST, AE
**MA**	**20**	46	F	Other	0	U	III	-	AG, ASA
**MA**	**21**	50	F	HS	1	B	IV	-	AD
**MA**	**23**	71	F	IS	7	U	III	DL	AG, ASA, ST, AE
**MA**	**24**	51	F	TIA	0	B	III	HoS, ET	AG, ASA, ST, AE
**MA**	**25**	54	F	Other	0	U	III	HoS	-
**MA**	**30**	30	F	TIA	-	U	III	-	AG, ASA
**MA**	**31**	49	M	IS	3	U	IV	DM, DL, HoS	AG, ASA, Other
**MA**	**33**	70	F	Other	0	U	-	H, DL, HoS	AG, ASA
**MA**	**34**	22	F	TIA	10	B	IV	H	-
**MA**	**35**	30	M	Other	0	B	V	DL, AA	-
**MA**	**37**	28	F	Other	0	U	IV	PI	AG
**MA**	**38**	63	M	HS	0	B	IV	H, DM, DL, IHH, HT	ST, CCB
**MA**	**40**	47	F	TIA	3	B	IV	H, HoS, PI	AG, ASA, Other
**MA**	**41**	52	F	Other	0	B	V	PI	-
**MA**	**42**	39	F	HS	0	B	III	DL,HoS, PI	ST
**MA**	**43**	45	F	IS	0	B	IV	PI, HHC	AG,ASA
**MA**	**45**	60	F	IS	4	B	V	H, DL	AG, ASA ST
**MA**	**48**	52	F	HS	0	U	IV	-	-
**MA**	**49**	36	F	Other	-	U	VI	PI, ET, HT	Other
**MA**	**51**	56	F	IS	0	B	III	H, DL, HoS, HHC	AG, ASA, ST
**MA**	**52**	37	F	IS	2	B	VI	H, DL, ET. HT	AG, ASA, ST, Other
**MA**	**53**	45	M	TIA	-	B	VI	DL, HoS, AA, HT	AG, ASA,ST
**MA**	**54**	53	M	TIA	0	U	IV	H, DL	AG, ASA, ST, Other
**MA**	**56**	53	F	TIA	-	B	-	-	AG, ASA, Other
**MA**	**58**	30	F	IS	5	B	III	HoS	AG, ASA
**MA**	**59**	56	F	TIA	0	B	IV-V	H, DL	AG, ASA, ST, AE
**MA**	**61**	38	F	TIA, Other	0-2	B	IV	HoS,PI	AG, ASA, Other
**MA**	**63**	53	F	HS	0	U	-	HT	ST, AE
**MA**	**66**	43	F	IS	2	U	-	HoS	AG, ST
**MA**	**68**	40	F	HS	-	U	-	HoS, PI	-
**MA**	**70**	27	F	Other	-	B	-	H, PI	AG, ASA, AE
**MA**	**71**	47	F	IS	4	B	-	H, HoS, PI	AG, ASA,Other

Supplementary Table S1: Demographic, clinical and neuro-radiological features of 40 MA patients whose plasma samples were included in biological analyses (AA, alcohol abuse; AD, antidepressant; AE, antiepileptic; AG, antiaggregants; AH, antihypertensive; AHHC, anti-hyperhomocysteinemia; ASA, acetylsalicylic acid; B, bilateral; CCB, calcium channel blockers; CVD, cerebrovascular disease; DL, dyslipidemia; DM, diabetes mellitus; ET, estroprogestinic therapy; f, female; H, hypertension; HHC, hyperhomocysteinemia; HoS, history of smoking; HS, hemorrhagic stroke; HT, head trauma; IHH, ischemic heart disease; IS, ischemic stroke; m, male; MA, moyamoya arteriopathy; NIHSS, National Institute of Health scale; PI, physical inactivity; PSY, psychiatric disorder; ST, statins; TIA, transient ischemic attack; U, unilateral).

ID		Age	Gender	CVD type	NIHSS	U-B	Suzuki grading	Vascular risk factors	Pharmacological therapy
**MA**	**5**	44	F	HS	5	B	IV	H	AE, AD
**MA**	**15**	47	F	IS	8	B	IV	H, PI,	AG, ASA, ST, AE
**MA**	**19**	42	F	IS	2	B	IV	HT	AG, ASA, ST, AE
**MA**	**21**	50	F	HS	1	B	IV	-	AD
**MA**	**24**	51	F	TIA	0	B	III	HoS, ET	AG, ASA, ST, AE
**MA**	**34**	22	F	TIA	10	B	IV	H	-
**MA**	**40**	47	F	TIA	3	B	IV	H, HoS, PI	AG, ASA, Other
**MA**	**41**	52	F	Other	0	B	V	PI	-
**MA**	**42**	39	F	HS	0	B	III	DL,HoS, PI	ST
**MA**	**43**	45	F	IS	0	B	IV	PI, HHC	AG,ASA
**MA**	**45**	60	F	IS	4	B	V	H, DL	AG, ASA ST
**MA**	**51**	56	F	IS	0	B	III	H, DL, HoS, HHC	AG, ASA, ST
**MA**	**52**	37	F	IS	2	B	VI	H, DL, ET. HT	AG, ASA, ST, Other
**MA**	**59**	56	F	TIA	0	B	IV-V	H, DL	AG, ASA, ST, AE
**MA**	**61**	38	F	TIA,Other	0-2	B	IV	HoS,PI	AG, ASA, Other

Supplementary Table S2: Demographic, clinical and neuroradiological features of 15 MA patients (AA, alcohol abuse; AD, antidepressant; AE, antiepileptic; AG, antiaggregants; AH, antihypertensive; AHHC, anti-hyperhomocysteinemia; ASA, acetylsalicylic acid; B, bilateral; CVD, cerebrovascular disease; DL, dyslipidemia; DM, diabetes mellitus; ET, estroprogestinic therapy; f, female; H, hypertension; HHC, hyperhomocysteinemia; HoS, history of smoking; HS, hemorrhagic stroke; HT, head trauma; IHH, ischemic heart disease; IS, ischemic stroke; m, male; MA, moyamoya arteriopathy; NIHSS, National Institute of Health scale; PI, physical inactivity; PSY, psychiatric disorder; ST, statins; TIA, transient ischemic attack; U, unilateral).

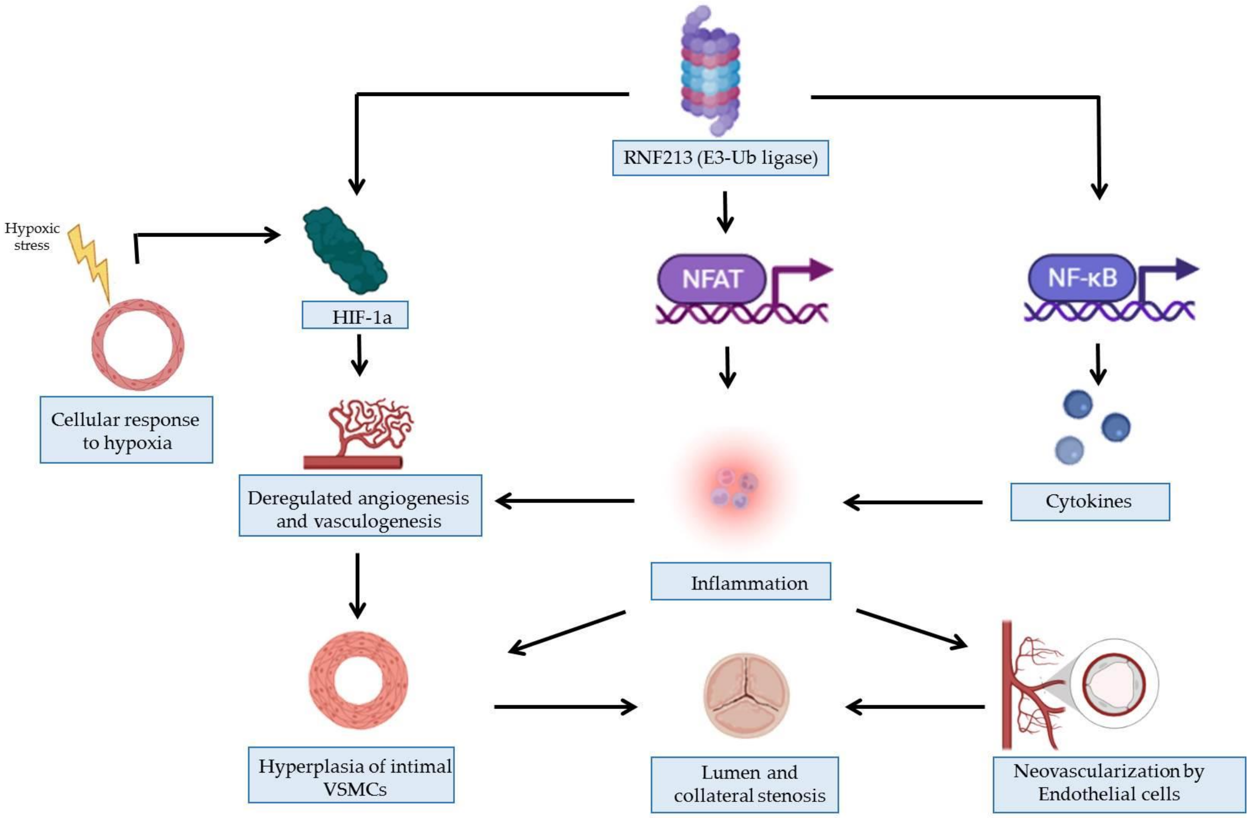

Supplementary Figure S1. Implications of RNF213 in vasculogenic/angiogenic/inflammatory pathways.

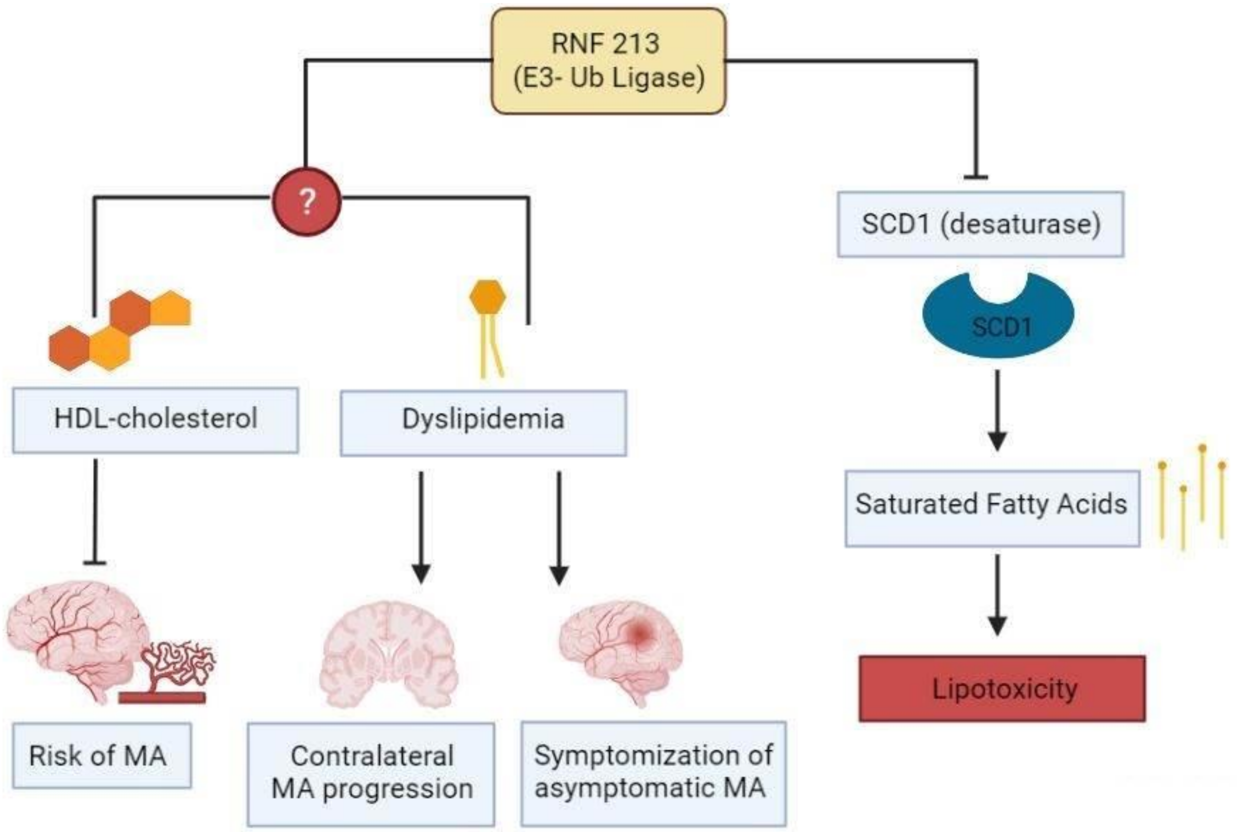

Supplementary Figure S2. Putative effects of RNF213 in lipid metabolism.
